# Dermoscopy: a useful tool for assisting the diagnosis of *Pseudomonas* folliculitis*

**DOI:** 10.1590/abd1806-4841.20165382

**Published:** 2016

**Authors:** Enzo Errichetti, Giuseppe Stinco

**Affiliations:** 1 University of Udine – Udine, Italy

**Keywords:** Dermoscopy, Diagnosis, differential, Pseudomonas infections

## Abstract

This report describes the usefulness of dermoscopy as a supportive diagnostic tool in a pseudomonas folliculitis case.

Pseudomonas folliculitis (PF) is a community-acquired infection, typically resulting from the bacterial colonization of hair follicles after direct exposure to contaminated water (e.g. in whirlpools, swimming pools, water slides and bathtubs), or the use of contaminated bathing objects (e.g. sponges and inflatable pool toys). However, obvious sources of contamination are not always detectable.^1^ Lesions usually appear on the skin within hours or days following the exposure and consist of pruritic, erythematous macules that progress to 2-10mm in diameter, and edematous papules, some of which have a follicle-centered pustule.^1^ This rash favors the intertriginous areas or sites covered by bathing suits and it usually fades away spontaneously within 2-10 days.^1^ PF is commonly mistaken for other disorders presenting with erythemato-edematous papules and, consequently, unnecessary therapies are frequently prescribed.^1^ This report describes the usefulness of dermoscopy as a supportive diagnostic tool in a PF case.

A 41-year-old Caucasian woman presented with a 5-day history of an itchy rash, localized mainly on her armpits, inguinal areas and thighs. Before coming to the clinic, she had been diagnosed with insect bites but topical steroid application had not entailed any improvement. The patient was otherwise healthy and was not taking any medication. Her past medical history was unremarkable and she could not recall any obvious recent exposures to potential *Pseudomonas aeruginosa* sources. Physical examination revealed numerous erythemato-edematous papules and a few pustules (Figures 1A and 1B). On polarized light noncontact dermoscopic examination (DermLite DL3 x10; 3Gen, San Juan Capistrano, CA, USA), all the papules exhibited a pinkish background with a paler centre and a central vellus hair, thus highlighting the folliculocentric nature of the rash; no distinct vessel was evident (Figure 1C). Swab cultures taken from the pustules were positive for *Pseudomonas aeruginosa*, thus confirming the diagnosis of PF. Gentamicin 0.1% cream (twice daily) was prescribed and lesions cleared after five days.

**Figure 1:**
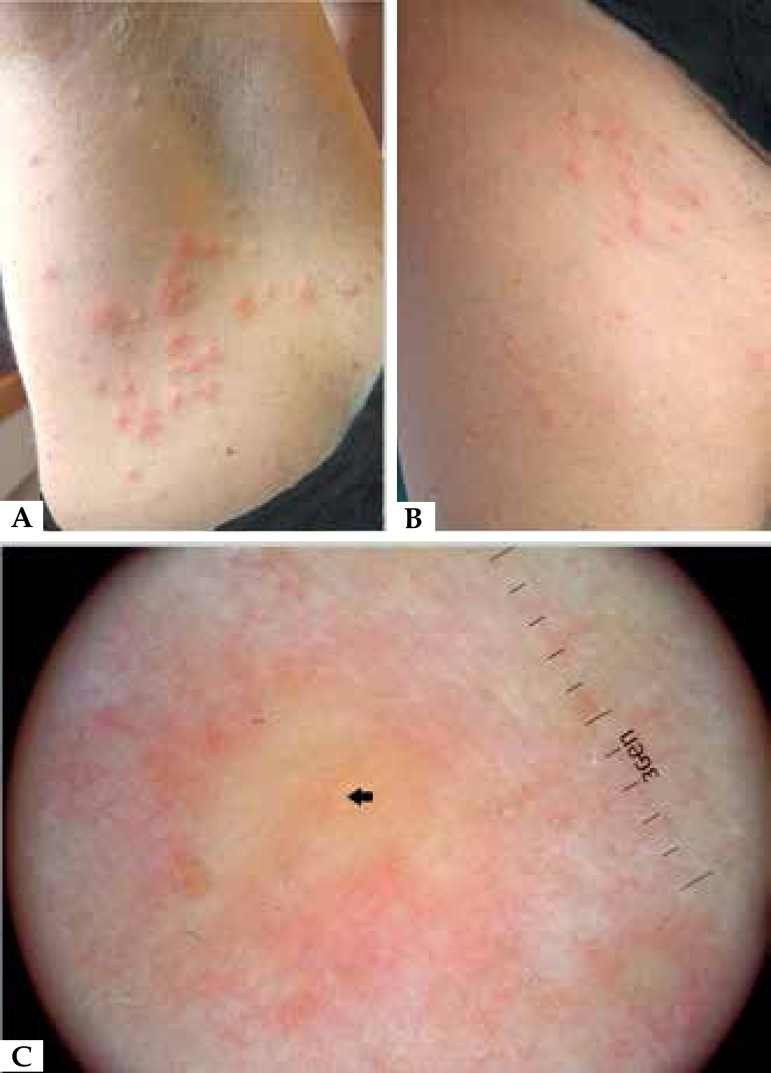
Physical examination revealing several erythemato-edematous papules and a few pustules on the right armpit (**a**) and inguinal area (**b**). Polarized light noncontact dermoscopic examination (X10 magnification) of a papule displays a pinkish background with a paler centre and a central thin vellus hair (black arrow); no distinct vessel is evident (**c**).

The main, challenging differential diagnoses for PF include insect bites and nodular scabies. ^1,2^ The distinction from such conditions is typically clinical, evidenced by the folliculocentric nature of the lesions and positive lesional swabs.^1,2^ However, detecting the former feature may be troublesome, particularly in subjects with fair skin/hair and when lesions are located on sites with few terminal hairs.

Dermoscopy is a low-cost, noninvasive technique that allows the clinician to note significant findings, which are not visible to the naked eye.^3-10^ In recent years, its use has been extended to numerous "general" dermatoses to assist clinical diagnosis. ^3-10^ In this PF case, dermoscopy proved helpful in identifying the vellus hairs at the centre of each lesion, otherwise not clinically visible, thus displaying the folliculocentric nature of the rash and therefore ruling out insect bites and nodular scabies. In fact, the lesions of these conditions are typically not centered around follicles and usually reveal other dermoscopic findings. In particular, insect bites may display a central punctum and some haemorrhagic spots (personal observations), while nodular scabies is generally characterized by mites ("hang glider sign") and/or burrows ("jet with condensation trails").^3^ Furthermore, in the authors' opinion, dermoscopy may be useful even to distinguish PF from staphylococcal folliculitis since, unlike the former, its lesions typically do not exhibit a central pale aspect (corresponding to the remarkable oedema present in PF) but display central pustules on a reddish background with or without nonspecific vessels.^1,6^

In conclusion, dermoscopy may be a useful tool for assisting the noninvasive diagnosis of some challenging PF cases. Further studies on larger groups of patients are needed to support the observations.
